# Factors associated with red blood cell transfusions in very-low-birth-weight preterm infants in Brazilian neonatal units

**DOI:** 10.1186/s12887-015-0432-6

**Published:** 2015-09-04

**Authors:** Amelia Miyashiro Nunes dos Santos, Ruth Guinsburg, Maria Fernanda Branco de Almeida, Renato Soibelman Procianoy, Sergio Tadeu Martins Marba, Walusa Assad Gonçalves Ferri, Ligia MariaSuppo de Souza Rugolo, José Maria Andrade Lopes, Maria Elisabeth Lopes Moreira, Jorge Hecker Luz, Maria Rafaela Conde González, Jucille do Amaral Meneses, Regina Vieira Cavalcante da Silva, Vânia Olivetti Steffen Abdallah, José Luiz Muniz Bandeira Duarte, Patricia Franco Marques, Maria Albertina Santiago Rego, Navantino Alves Filho, Vera Lúcia Jornada Krebs, For the Brazilian Network on Neonatal Research

**Affiliations:** Universidade Federal de São Paulo, São Paulo, SP Brazil; Universidade Federal do Rio Grande do Sul, Porto Alegre, RS Brazil; Universidade Estadual de Campinas, Campinas, SP Brazil; Universidade de São Paulo, Ribeirão Preto, SP Brazil; Universidade Estadual Paulista Júlio de Mesquita Filho, Botucatu, SP Brazil; Instituto Nacional de Saúde da Mulher, Criança e Adolescente Fernandes Figueira - Fundação Oswaldo Cruz, Avenida Rui Barbosa, 716, Rio de Janeiro, RJ, CEP 22420040 Brazil; Pontifícia Universidade Católica do Rio Grande do Sul, Porto Alegre, RS Brazil; Universidade Estadual de Londrina, Londrina, PR Brazil; Instituto de Medicina Integral Professor Fernando Figueira, Recife, PE Brazil; Universidade Federal do Paraná, Curitiba, PR Brazil; Universidade Federal de Uberlândia, Belo Horizonte, MG Brazil; Universidade do Estado de Rio de Janeiro, Rio de Janeiro, RJ Brazil; Universidade Federal do Maranhão, São Luís, MA Brazil; Universidade Federal de Minas Gerais, Belo Horizonte, MG Brazil; Faculdade de Ciências Médicas de Minas Gerais, Belo Horizonte, MG Brazil; Universidade de São Paulo, São Paulo, SP Brazil

**Keywords:** Very low birth weight infants, Neonatal intensive care unit, Anemia, Red blood cell transfusion, Risk factors

## Abstract

**Background:**

Preterm infants in neonatal intensive care units frequently receive red blood cells (RBC) transfusions due to the anemia of prematurity. A number of variables related to gestational age, severity of illness and transfusion practices adopted in the neonatal unit where the neonate was born may contribute to the prescription of RBC transfusions. This study aimed to analyse the frequency and factors associated with RBC transfusions in very-low-birth-weight preterm infants.

**Methods:**

A prospective cohort of 4283 preterm infants (gestational age: 29.9 ± 2.9 weeks; birth weight: 1084 ± 275 g) carried out at 16 university hospitals in Brazil between January 2009 and December 2011 was analysed. Factors associated with RBC transfusions were evaluated using univariate and multiple logistic regression analysis.

**Results:**

A total of 2208 (51.6 %) infants received RBC transfusions (variation per neonatal unit: 34.1 % to 66.4 %). RBC transfusions were significantly associated with gestational age (OR: -1.098; 95%CI: -1.12 to -1.04), SNAPPE II score (1.01; 1.00-1.02), apnea (1.69; 1.34-2.14), pulmonary hemorrhage (2.65; 1.74-4.031), need for oxygen at 28 days of life (1.56; 1.17-2.08), clinical sepsis (3.22; 2.55-4.05), necrotising enterocolitis (3.80; 2.26-6.41), grades III/IV intraventricular hemorrhage (1.64; 1.05-2.58), mechanical ventilation (2.27; 1.74-2.97), use of umbilical catheter (1.86; 1.35-2.57), parenteral nutrition (2.06; 1.27-3.33), >60 days of hospitalization (5.29; 4.02-6.95) and the neonatal unit where the neonate was born.

**Conclusions:**

The frequency of RBC transfusions varied among neonatal intensive care units. Even after adjusting for adverse health conditions and therapeutic interventions, the neonatal unit continued to influence transfusion practices in very-low birth-weight infants.

## Background

Preterm infants are at greater risk of developing anemia in comparison to full-term newborns, especially when admitted to an intensive care unit [1, 2]. Red blood cell (RBC) transfusions are one of the most employed strategies to correct anemia in preterm infants and have been associated with adverse health conditions and death, especially among preterm infants [3–6].

Understanding the events that increase the likelihood of RBC transfusions can contribute to the rational use of blood products in very low birth weight infants. Literature reports that RBC transfusions in preterm neonates are associated with clinical severity, phlebotomy blood loss and use of liberal criteria for the indication of transfusions. Among 640 newborns with a mean birth weight of 880 g and gestational age of 26 weeks, Fabres et al. found that 85 % of them received at least one red blood cell transfusion and that the transfusion volume was associated with birth weight, gestational age, age at first transfusion and the use of inotropic drugs [7]. Analyzing 147 newborns with a gestational age of 23.6 to 35.7 weeks and a birth weight of 460 to 1495 g, Mimica et al. found that number of transfusions was associated with birth weight, phlebotomy blood loss, duration of mechanical ventilation, peri-intraventricular hemorrhage and the use of liberal RBC transfusion guidelines. Every 10 mL/kg of blood lost increased the number of transfusions by 0.66; every 10 days on mechanical ventilation increased the number of transfusion by 0.59; and the adoption of liberal criteria increased this number by 0.55 [1]. A multicenter study carried out in seven neonatal units found that phlebotomy blood loss of 10 mL/kg increased the number of RBC transfusions by 27 % (95 % confidence interval: 23 to 30 %).

In this context, the aim of the present study was to analyze the frequency of red blood cell transfusions in very-low-birth-weight preterm infants and factors associated with this procedure in neonatal intensive care units in Brazil.

## Methods

Data collected prospectively from 16 neonatal intensive care units at university hospitals located in seven states in Brazil were analyzed for this study. At each unit, clinical maternal and neonatal data considered essential to improvements in the quality of clinical care were routinely collected using a specific chart. At the time of the data collection for this study, delayed cord clamping or cord milking was not a routine practice at these neonatal units. Each unit was identified with a different letter of the alphabet (A to P).

The research project was approved by the Research Ethics Committee of each of the 16 network units (Research Ethics Committee of Instituto Fernandes Figueira - *Fundação Oswaldo Cruz*, Universidade Federal de São Paulo, Universidade de São Paulo, Universidade de Campinas, Universidade Federal do Rio Grande do Sul, Universidade de São Paulo, Ribeirão Preto, Universidade Estadual Paulista Júlio de Mesquita Filho, Pontifícia Universidade Católica do Rio Grande do Sul, Universidade Estadual de Londrina, Instituto de Medicina Integral Professor Fernando Figueira, Universidade Federal de Uberlândia, Universidade do Estado de Rio de Janeiro, Universidade Federal do Maranhão, Universidade Federal de Minas Gerais, Faculdade de Ciências Médicas de Minas Gerais). The Research Ethics Committee of Instituto Fernandes Figueira - *Fundação Oswaldo Cruz*, Rio de Janeiro was the leading center for database evaluation in the network (CAAE: # 12244913.2.0000.5505). Since data was obtained from a prospective database of all infants born in the Brazilian Network on Neonatal Research in the study period, informed consent was not obtained from participants because it is not required according to Brazilian regulations and IRB approval. The data were used for the analysis of transfusion frequencies and factors associated with the indication for RBC transfusions in very-low-birth-weight newborns. The inclusion criteria were gestational age 22 to 36.9 weeks, birth weight < 1500 g and births occurring in the 16 neonatal units of the Brazilian Network on Neonatal Research. Infants with malformations and those who died in the first 12 h of life were excluded from the study.

The following factors were investigated: mother’s clinical and obstetric history; birth conditions; sex; gestational age (calculated from the best obstetric estimate or by the neonatal evaluation) [8]; adequacy of birth weight to gestational age [9]; Apgar scores; clinical severity in the first 12 h of life based on the Score for Neonatal Acute Physiology, Perinatal Extension, Version II (SNAPPE II) [10]; neonatal morbidity; and RBC transfusions during hospital stay. The following clinical morbidities were analyzed: respiratory distress syndrome [11]; pulmonary hemorrhage; apnea [12]; dependence on oxygen at 28 days of life and/or at 36 weeks of corrected gestational age [13]; patent ductus arteriosus [14]; clinical sepsis [15]; necrotizing enterocolitis [16]; peri-intraventricular hemorrhage (diagnosed by at least one head ultrasound during hospital stay) [17]; periventricular leukomalacia [18]; and retinopathy of prematurity according to The International Classification of Retinopathy of Prematurity [19]. Data were also collected on the use of mechanical ventilation, vasoactive drugs, umbilical catheter placement and parenteral nutrition.

**Fig. 1:**
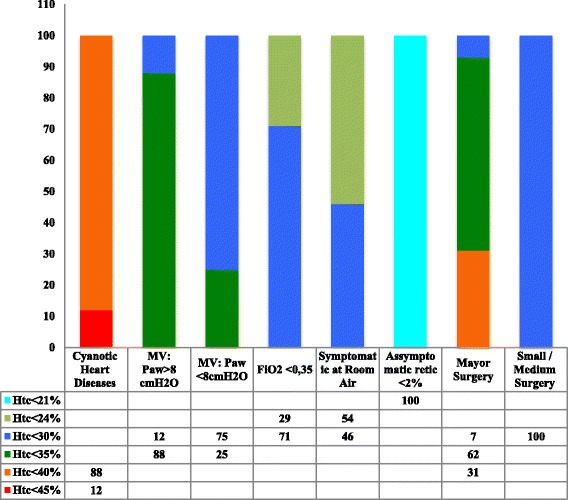
Percentage of neonatal units that indicate red blood cell transfusions when hematocrit is lower than 21 %, 24 %, 30 %, 35 %, 40 % or 45 %, according to clinical characteristics of newborns and need for respiratory support or surgery. MV: mechanical ventilation; MAP: mean airway pressure; FiO_2_: inspired oxygen fraction; retic: reticulocytes count

All neonatal units had written guidelines for RBC transfusions based on the clinical condition of the newborn and on the need for respiratory support or surgery (Fig. 1). Four units employed criteria based on the chronological age of the newborn. At these units, transfusions in the first two weeks were indicated when the hematocrit was lower than 35 to 40 % in the presence of respiratory support or surgery need, symptoms of anemia or hematocrit lower than 25 to 35 % in asymptomatic infants. Beginning in Week 3, RBC transfusions were indicated when the hematocrit was lower than 25 to 30 % in symptomatic infants or if infants needed respiratory support or surgery or hematocrit was lower than 21 % in asymptomatic infants. The transfusion volume ranged from 10 to 20 mL/kg; 47 % of the neonatal units administered 15 mL/kg at each transfusion.

The convenience sample was composed of all very-low-birth-weight newborns who met the eligibility criteria. According to literature [20], 15 patients are needed for each independent variable in the logistic regression model. Considering the 35 independent variables analyzed in the present study (19 clinical and 16 neonatal units), a minimum of 525 patients was needed for the present study.

Univariate analysis was performed to test the strength of associations between the independent variables and the occurrence of RBC transfusions, with the inclusion of clinical variables considered important to the event. Variables with a p-value < 0.20 were incorporated in the multiple logistic regression analysis and those with a p-value < 0.05 remained in the final model. Statistical analysis was performed with the software SPSS 17.0 (*IBM SPSS Statistics,* Somers, NY), considering significant, p < 0.05.

## Results

A total of 4882 neonates with very-low-birth-weight (gestational age: 22 to 36.9 weeks) were admitted to the 16 neonatal units during the study period. A total of 599 were excluded based on the eligibility criteria. Thus, 4283 were included in the study (mean gestational age: 29.9 ± 2.9 weeks: birth weight: 1084 ± 275 g); 2187 (51.1 %) were male; and 2937 (68.6 %) were delivered by cesarean section. Apgar scores were 5.8 ± 2.5 at the first minute and 8.0 ± 1.7 at the fifth minute. The SNAPPE II score was 24 ± 24. A total of 2330 (54.4 %) required positive-pressure ventilation in the delivery room.

During the hospital stay in the neonatal intensive care unit, the following complications were recorded: respiratory distress syndrome (n = 2755; 64.3 %), pulmonary hemorrhage (n = 262; 6.1 %), persistent arterial duct (n = 1443; 33.7 %), apnea (n = 1826; 42.6 %), oxygen dependence at 28 days of life (n = 1086; 25.4 %), oxygen dependence at a corrected gestational age of 36 weeks (n = 995; 23.2 %), clinical sepsis (n = 2563; 59.8 %), necrotizing enterocolitis (n = 307; 7.2 %), any degree of peri-intraventricular hemorrhage (n = 1101; 25.7 %), grade III or IV intraventricular hemorrhage (n = 325; 7.6 %), periventricular leukomalacia (n = 264; 6.2 %) and retinopathy of prematurity (n = 787; 18.4 %). Moreover, 2615 (61.1 %) neonates were submitted to mechanical ventilation (median duration: 6 days; Q1-Q3: 2-16 days), 2757 (64.4 %) received an umbilical catheter, 465 (10.9 %) received vasoactive drugs and 3802 (88.8 %) received parenteral nutrition (median duration: 11 days; Q1-Q3: 7-17 days). Median stay in the neonatal intensive care unit was 42 days (Q1-Q3: 26-64 days).

A total of 2208 (51.6 %) newborns received RBC transfusions. Table 1 displays the frequency of transfusions at each neonatal unit analyzed. Table 2 shows the characteristics of the newborns who received transfusions and those who did not.

**Table 1:** Number and percentage of infants transfused in each neonatal unit

Neonatal units	Included neonates	Transfused neonates	Percentage
A	168	68	40.5
B	881	394	44.7
C	249	148	59.4
D	153	69	45.1
E	303	161	53.1
F	217	74	34.1
G	334	190	56.9
H	226	150	66.4
I	263	131	49.8
J	122	52	42.6
K	343	223	65.0
L	227	143	63.0
M	273	94	34.4
N	181	130	71.8
O	134	69	51.5
P	210	112	53.3
Total	4283	2208	51.6

**Table 2:** Clinical characteristics of transfused vs. not transfused infants

	Transfused (n = 2208)	Non-transfused (n = 2075)	*p*
Gestational age (weeks)	28.8 ± 2.6	31.1 ± 2.7	<0.001
Gestational age <28 weeks [n (%)]	798 (36.1 %)	230 (11.1 %)	<0.001
Birth weight (grams)	971 ± 254	1205 ± 242	<0.001
Birth weight <1000 grams [n (%)]	1211 (54.8 %)	380 (18.3 %)	<0.001
5^th^ minute Apgar <7 [n (%)]	428 (19.4 %)	184 (8.9 %)	<0.001
SNAPPE II (score)	30 ± 22	18 ± 18	<0.001
Respiratory distress syndrome [n (%)]	1749 (79.2 %)	1006 (48.5 %)	<0.001
Apnea [n (%)]	1168 (52.9 %)	658 (31.7 %)	<0.001
Pulmonary hemorrhage [n (%)]	216 (9.8 %)	46 (2.2 %)	<0.001
Patent ductus arteriosus [n (%)]	955 (43.3 %)	488 (23.5 %)	<0.001
Clinical sepsis [n (%)]	1808 (81.9 %)	755 (36.4 %)	<0.001
Necrotizing enterocolitis [n (%)]	266 (12.0 %)	41 (2.0 %)	<0.001
PIVH grade 3-4 [n (%)]	240 (10.9 %)	85 (4.1 %)	<0.001
Periventricular leukomalacia [n (%)]	183 (8.3 %)	81 (3.9 %)	<0.001
Retinophaty of prematurity [n (%)]	514 (23.3 %)	273 (13.2 %)	<0.001
Need for oxygen at 28 days of life [n (%)]	799 (36.2 %)	287 (13.8 %)	<0.001
Need for O_2_ therapy at 36 weeks [n (%)]	485 (22.0 %)	153 (7.4 %)	<0.001
Use of umbilical catheter [n (%)]	1677 (76.0 %)	1080 (52.0 %)	<0.001
Need for mechanical ventilation [n (%)]	1848 (83.7 %)	767 (37.0 %)	<0.001
Duration of mechanical ventilation (days)	13 ± 19	3 ± 8	<0.001
Need for vasoactive drugs [n (%)]	328 (14.9 %)	119 (5.7 %)	<0.001
Use of paraenteral nutrition [n (%)]	2136 (96.7 %)	1666 (80.3 %)	<0.001
Duration of hospitalization (days)	59 ± 41	35 ± 20	<0.001
Hospitalization > 60 days [n (%)]	1053 (47.7 %)	184 (8.9 %)	<0.001
Intra-hospital death [n (%)]	662 (30.0 %)	300 (14.5 %)	<0.001

*SNAPPE II* Morbidity and mortality risk scores, *PIVH* peri-intraventricular hemorrhage

The univariate logistic regression to analyze factors associated with the use of RBC transfusions is shown in Table 3. Each neonatal unit was compared to Unit F, which had the lowest rate of RBC transfusions (Table 3). Multiple logistic regression analysis was then performed in order to determine the odd ratios for receiving RBC transfusions (Table 4).

**Table 3:** Unadjusted odds ratio of receiving RBC transfusions according to infant’s characteristics and neonatal unit

	OR	95 % CI	*p*
Gestational age (weeks)	0.731	0.713 – 0.750	<0.001
Birth weight < 1000 grams	5.418	4.714 – 6.228	<0.001
5^th^ minute Apgar <7	2.472	2.055 – 2.973	<0.001
SNAPPE II (score)	1.033	1.029 – 1.037	<0.001
Respiratory distress syndrome	4.049	3.541 – 4.630	<0.001
Apnea	2.419	2.135 – 2.740	<0.001
Patent ductus arteriosus	2.479	2.172 – 2.828	<0.001
Clinical sepsis	7.903	6.867 – 9.094	<0.001
Necrotizing enterocolitis	6.795	4.862 – 9.496	<0.001
PIVH grades 3-4	2.855	2.212 – 3.685	<0.001
Need for oxygen at 28 days of life	1.625	1.502 – 1.757	<0.001
Retinophaty of prematurity	2.003	1.705 – 2.353	<0.001
Use of umbilical catheter	2.910	2.555 – 3.314	<0.001
Need for mechanical ventilation	8.754	7.581 – 10.109	<0.001
Use of vasoactive drugs	2.868	2.304 – 3.570	<0.001
Need for parenteral nutrition	7.870	6.009 – 10.308	<0.001
Intra-hospital death	2.534	2.135 – 2.740	<0.001
Unit M	1.015	0.697 – 1.477	0.939
Unit A	1.341	0.883 – 2.036	0.169
Unit J	1.436	0.910 – 2.264	0.120
Unit B	1.563	1.146 – 2.133	0.005
Unit D	1.587	1.038 – 2.427	0.033
Unit I	1.918	1.324 – 2.778	0.001
Unit O	2.051	1.321 – 3.185	0.001
Unit E	2.176	1.518 – 3.118	<0.001
Unit P	2.208	1.495 – 3.263	<0.001
Unit G	2550	1.789 – 3.635	<0.001
Unit C	2.832	1.941 – 4.132	<0.001
Unit L	3.290	2.229 – 4.854	<0.001
Unit K	3.591	2.511 – 5.136	<0.001
Unit H	3.814	2.573 – 5.654	<0.001
Unit N	4.926	3.209 – 7.561	<0.001

*SNAPPE II* Morbidity and mortality risk scores, *PIVH* peri-intraventricular hemorrhage

**Table 4:** Final model of multiple logistic regression for factors associated with RBC transfusions

	OR	IC 95 %	*p*
Gestational age (weeks)	-1.098	-1.151 – -1.042	<0.001
SNAPPE II	1.011	1.004 – 1.017	0.001
Apnea	1.692	1.339 – 2.138	<0.001
Pulmonary hemorrhage	2.648	1.740 – 4.031	<0.001
Clinical sepsis	3.217	2.553 - 4.054	<0.001
PIVH grades 3-4	1.642	1.046 -2.576	0.031
Necrotizing enterocolitis	3.804	2.258 – 6.407	<0.001
Need for oxygen at 28 days of life	1.562	1.173 – 2.081	0.002
Use of umbilical catheter	1.864	1.352 – 2.569	<0.001
Need for mechanical ventilation	2.271	1.740 – 2.966	<0.001
Use of parenteral nutrition	2.058	1.271 – 3.332	0.003
Hospitalisation >60 days	5.286	4.020 – 6.949	<0.001
Unit B	2.246	1.156 – 4.365	0.017
Unit O	2.822	1.230 – 6.470	0.014
Unit I	2.887	1.383 – 6.026	0.005
Unit P	2.961	1.365 – 6.423	0.006
Unit E	4.790	2.434 – 9.427	<0.001
Unit H	5.638	2.729 – 11.651	<0.001
Unit C	6.120	2.991 – 12.523	<0.001
Unit N	6.248	2.840 – 13.740	<0.001
Unit L	8.120	3.934 – 16.759	<0.001
Unit K	8.396	4.376 – 16.111	<0.001
Unit G	8.434	4.187 – 16.989	<0.001

Significance of the model p < 0.001. Final model adjusted for gestacional age, *SNAPPE II* respiratory distress syndrome, apnea, pulmonar hemorrhage, patente ductus arteriosus, clinical sepsis, necrotizing enterocolitis, need for oxygen therapy at 28 days of life, peri-intraventricular hemorrhage grades 3-4 (PIVH 3-4), retinopathy of prematurity, use of umbilical cateter, need for mechanical ventilation, use of parenteral nutrition, hospitalization > 60 days, and neonatal unit where the neonate was born

## Discussion

Considerable variability among neonatal intensive care units was found regarding the frequency of RBC transfusions. The following factors were associated with indications for transfusions: lower gestational age, higher SNAPPE II score, presence of apnea, pulmonary hemorrhage, clinical sepsis, moderate/severe intraventricular hemorrhage, necrotizing enterocolitis, bronchopulmonary dysplasia, umbilical catheter use, parenteral nutrition, prolonged hospital stay and center. These variables are closely interrelated and, to some extent, reflect the morbidities and procedures often described for very-low-birth-weight newborns. However, the cross-sectional design of this investigation does not allow the determination of cause-and-effect relationships between these variables and the transfusion of RBC [21].

Variability in the frequency of RBC transfusions is often reported in literature [7, 22–24]. This variability may be due to the increasing survival rate of premature infants with lower gestational ages [25], but is also likely due to a lack of evidence-based criteria for the indication of transfusions [26]. Thus, the transfusion rate varies among neonatal units due to the severity of the patients’ condition. In the present study, patients with higher SNAPPE II score had higher likelihood of being transfused. SNAPPE II score is related with mortality and morbidity [10] and may be associated with red blood cell transfusions [4–7]. Kling et al. described a prediction model for transfusion in preterm neonates based on phlebotomy blood loss and clinical severity score, even after adjusting for phlebotomy blood loss [27].

The restrictiveness of the transfusion guidelines may also contribute to the transfusion rates. A study carried out in 11 countries involving interviews with 1018 neonatologists found that only 51.1 % of the neonatal units have written guidelines for the indication of transfusions [28].

A number of studies report an increase in adverse health conditions associated with RBC transfusions in premature infants, such as necrotizing enterocolitis [5, 29–31], ventricular hemorrhage [3, 29] and death [4]. In a retrospective study involving 417 premature infants with grade I peri-intraventricular hemorrhage, 24 developed grade III hemorrhage and 22 developed grade IV hemorrhage after RBC transfusions. The factors associated with the progression in hemorrhage severity were gestational age (OR: 0.95; 95 % CI: 0.92 to 0.98) and having received a transfusion (OR: 2.92; 95 % CI: 2.19 to 3.90) [3]. In a meta-analysis that included retrospective and case-control studies involving 4857 premature infants, Mohamed and Shah found an association between RBC transfusion and the occurrence of necrotizing enterocolitis 48 h following the transfusion. After controlling for confounding factors, the odds of developing enterocolitis was 2.01-fold (95 % CI: 1.61-2.50) greater among the infants that received transfusions in the previous 48 h in comparison to those who had not received transfusions [5]. Del Vecchio et al. described an association between the reduction of transfusion rates and a lower incidence of bronchopulmonary dysplasia, retinopathy of prematurity and necrotizing enterocolitis [32].

In the present study, premature newborns who received RBC transfusions during hospital stay had a 3.8-fold greater chance of developing enterocolitis and a 64 % greater chance of also having a diagnosis of grade III to IV hemorrhage in comparison to those who did not receive transfusions. However, it was not possible to establish the temporal relationship between the transfusions and these complications.

In a multicenter study involving tertiary neonatal care units at university hospitals and a total of 1077 premature newborns (gestational age: 23.0 to 36.9 weeks; birth weight: 400 to 1495 g), the relative risk of hospital death was 49 % greater among those who received at least one RBC transfusion in the first 28 days of life in comparison to those who did not receive transfusions, after controlling for confounding factors. Moreover, the relative risk of death after 28 days of life was 89 % greater among newborns who received three or more RBC transfusions during hospital stay in comparison to those who received one or two transfusions [4]. While this association was not investigated in the present study, it is a cause of concern that half of the newborns analyzed received at least one transfusion.

The use of an umbilical catheter was associated with a greater frequency of RBC transfusions. This association may have been due to the fact that umbilical catheters are employed in premature infants with lower gestational ages and greater clinical severity. However, an umbilical catheter facilitates blood collection for laboratory exams and can lead to greater blood loss, thereby increasing the need for transfusions.

The use of supplementary oxygen at 28 days of life, mechanical ventilation and vasoactive drugs were also associated with the indication for transfusions possibly due to the need for support to improve oxygenation and/or tissue perfusion. In a study by Guillén et al. involving 1018 neonatologists, the authors found that the following variables had the greatest influence on the decision to submit very-low-birth-weight newborns to RBC transfusions: the need for supplementary oxygen (44.7 % of neonatologists), need for respiratory support (44.1 %), postnatal age (36.5 %), number of reticulocytes (32.7 %) and the use of inotropic drugs (30.9 %). These findings suggest that the variables employed in the majority of guidelines for the indication of transfusions are effectively used in the clinical practice [28].

The variability in transfusion rates among the different neonatal units studied may be explained by the clinical diversity of the premature newborns cared at these services. However, the differences among units persisted even after adjusting for clinical variables considered risk factors for blood transfusions. It is therefore possible that several factors influenced the indication for transfusions at these neonatal units, such as phlebotomy blood loss, differences in the criteria employed for the indication of transfusions, the degree of compliance with existing protocols and other healthcare practices [33].

A reduction in phlebotomy blood loss is recognized as the most effective measure for diminishing the need for RBC transfusions. Madan et al. showed a 46 % reduction in the number of transfusions in extremely-low-birth-weight preterm infants using a bedside blood gas analyzer, which reduced the volume of blood for laboratory exams [34]. Mahieu et al. found a reduction in the percentage of premature newborns that received transfusions after adopting of a multi-parameter monitor for laboratory analyses (50.0 % to 38.9 %, p < 0.05) and a 38 % reduction in the number of transfusions per newborn (2.53 to 1.57; p < 0.01) [35].

The adoption of restrictive guidelines for RBC transfusions constitutes another measure for reducing the number transfusions. Venâncio et al. found that the use of restrictive criteria led to a reduction of 16 mL/kg in the volume of RBC transfused per newborn [36] and an 18 % reduction in the number of transfusions [1]. These effects are superior to those obtained with erythropoietin [2]. The practice of delayed cord clamping or cord milking would furher reduce the need for erythrocyte transfusions [37], but this practice is not routinely stablished in most neonatal units [28], as well as in the Brazilian neonatal units.

The limitations of our study were its: cross sectional design, lack of data on phlebotomy blood loss and number of transfusions per infant, and absence of data regarding transfusion guideline adherence in each neonatal unit. However the great number of neonates included and the diversity of factors analyzed may have contributed to improve the internal and external validity of this study.

## Conclusion

In conclusion, considerable variability was found among the different neonatal units studied regarding the frequency of RBC transfusions. Moreover, the influence of the center regarding transfusion practices in very-low-birth-weight newborns persisted even after controlling for confounding factors. The demographic and clinical characteristics of the patients, especially those related to clinical severity and the need for invasive procedures, were significantly associated with the indication for RBC transfusions in very-low-birth-weight newborns.
